# Identification and Validation of a Necroptosis-Related Prognostic Signature for Kidney Renal Clear Cell Carcinoma

**DOI:** 10.1155/2023/8446765

**Published:** 2023-03-03

**Authors:** Manbo Cai, Qiao Yang, Junyan He, Haibiao Wu, Zhimin Li, Zhe Fang, Jianjun Li

**Affiliations:** ^1^Department of Oncology Radiotherapy, The First Affiliated Hospital, Hengyang Medical School, University of South China, Hengyang City, China 421001; ^2^Department of Urological Surgical, The Second Affiliated Hospital, Hengyang Medical School, University of South China, Hengyang City, China 421001

## Abstract

**Background:**

Necroptosis is progressively becoming an important focus of research because of its role in the pathogenesis of cancer and other inflammatory diseases. Our study is designed to anticipate the survival time of kidney renal clear cell carcinoma (KIRC) by constructing a prognostic signature of necroptosis-related genes.

**Materials:**

Clinical information and RNA-seq data were acquired from Renal Cell Cancer-European Union (RECA-EU) and The Cancer Genome Atlas- (TCGA-) KIRC, respectively. ConsensusClusterPlus was used to identify molecular subtypes, and the distribution of immune cell infiltration, anticancer drug sensitivity, and somatic gene mutations was studied in these subtypes. Subsequently, LASSO-Cox regression and univariate Cox regression were also carried out to construct a necroptosis-related signature. Cox regression, survival analysis, clinicopathological characteristic correlation analysis, nomogram, cancer stem cell analysis, and receiver operating characteristic (ROC) curve were some tools employed to study the prognostic power of the signature.

**Results:**

Based on the expression patterns of 66 survival-related necroptosis genes, we classified the KIRC into three subtypes (C1, C2, and C3) that are associated with necroptosis, which had significantly different tumor stem cell components. Among these, C2 patients had a longer survival time and enhanced immune status and were more sensitive to conventional chemotherapeutic drugs. Moreover, in order to predict the prognosis of KIRC patients, five genes (BMP8A, TLCD1, CLGN, GDF7, and RARB) were used to develop a necroptosis-related prognostic signature, which had an acceptable predictive potency. The results from Cox regression and stratified survival analysis revealed that the signature was an independent prognostic factor, whereas the nomogram and calibration curve demonstrated satisfactory survival time prediction based on the risk score.

**Conclusions:**

Three molecular subtypes and five necroptosis-related genes were discovered in KIRC using data from TCGA-KIRC and RECA-EU. Thus, a new biomarker and a potentially effective therapeutic approach for KIRC patients were provided in the current study.

## 1. Introduction

One of the most common malignant tumors of the human urinary system is renal cell carcinoma (RCC), which accounts for about 80% of all types of renal cancer [1]. Its incidence is ranked sixth among male malignant tumors and tenth in female malignant tumors [2]. Kidney renal clear cell carcinoma (KIRC), the most prevalent and malignant kind of RCC, accounts for 70-80% of all renal cancers [3]. Hence, it is crucial to investigate the onset and progression of renal clear cell carcinoma. In recent years, because of the rapid progress of life science, scientists are increasingly exploring the genetic characteristics of this disease and exploring possible biological markers, which will provide a better basis for the diagnosis and treatment of KIRC. The development of a tailored treatment approach through reasonable combination and sequencing to provide patients with the best clinical efficacy is still a problem to be solved urgently though.

Necroptosis is a novel controlled type of cell death. According to Wang et al., receptor-interacting protein kinase 3 (RIPK3) and receptor-interacting protein kinase 1 (RIPK1) regulated programmed necrosis [4]. The distinct features of necroptosis cells include decreased ATP content, swollen mitochondria, elevated free radicals and intracellular calcium Ca2+, and infiltration of inflammatory factors [5, 6], accompanied by the release of damage-related molecular patterns that trigger *in vivo* inflammatory responses [7, 8]. Studies have shown that in numerous cancer cell lines, the expression of RIP3 is reduced or completely absent [9, 10]. In a cohort study involving more than 100 patients with colorectal cancer, low expression of RIP3 was found to independently predict reduced disease-free survival (DFS) and overall survival (OS) in patients [11]. Moreover, RIP3 knockout mice have demonstrated a higher risk of colitis-associated colorectal cancer and produce more promoters that are associated with tumors [12]. Epigenetic changes promote the downregulation of RIP1 expression and enhance the metastatic ability of tumor cells, resulting in the stimulated onset of squamous cell carcinoma of the head and neck [13]. All these studies suggest the antitumor role of programmed necrosis in cancer.

Cancer genomes provide a durable record of the genetic alterations that accumulate during carcinogenesis stemming from DNA damage and DNA repair defects. Copy number burden scores frac_altered and n_segs (“fraction altered” and “number of segments,” respectively) represent the fraction of bases deviating from baseline ploidy (defined as above 0.1 or below -0.1 in log_2_ relative copy number space) and the total number of segments in each sample's copy number profile, respectively [14]. Homologous recombination defect score is a measure quantifying defects in homologous recombination that sums 3 separate metrics of genomic scarring [15]. Another biomarker that has recently garnered significant attention is tumor mutational burden (TMB), which is a measure of the number of mutations in a cancer [16].

Currently, few studies systematically explore the association between necroptosis and KIRC. In the present paper, our team highlighted the expression profile of necroptosis in KIRC and their prognostic value through bioinformatics analysis. In order to accurately predict the prognosis of individuals with KIRC, we aimed to construct and validate a prognosis signature based on necroptosis. Additionally, our team investigated the link between the immunological microenvironment and somatic mutation and the prognostic characteristics of KIRC, which offers a theoretical basis for treatment regimens.

## 2. Material and Methods

### 2.1. Raw Data and Necroptosis-Related Genes

Clinical information and RNA-seq data were obtained from Renal Cell Cancer-European Union (RECA-EU) dataset and The Cancer Genome Atlas-kidney renal clear cell carcinoma (TCGA-KIRC) dataset, respectively. In addition, the International Cancer Genome Consortium (ICGC) (https://dcc.icgc.org/) and TCGA-Genomic Data Commons- (GDC-) API were retrieved. 74 necroptosis-related genes were acquired from previously finished topic research works [17].

For TCGA-KIRC and ICGC dataset, samples with clinical information, survival time, and status were remained. Ensembl was converted into gene symbol. The expression of multiple gene symbols was taken as the median value. In TCGA-KIRC dataset, genes whose expression was below 0.5 in the sample accounted for more than 50% were filtered.

### 2.2. Cluster Analysis

Firstly, the necroptosis score was calculated in TCGA-KIRC and RECA-EU using ssGSEA, and then, as per the standards of *R*| > 0.5 and *p* < 0.05, genes positively associated with necroptosis scores were determined. Next, the above genes were studied using the univariable Cox analysis through the Coxph function of R package survival in the TCGA-KIRC and RECA-EU dataset, and *p* < 0.05 was considered the liminal value. Finally, the intersection genes between the two datasets were retained. Afterward, molecular typing was performed separately for TCGA-KIRC dataset samples via the R package ConsensusClusterPlus 1.52.0 [18]. Pam arithmetic and “Canberra” distance were utilized to complete 500 bootstraps with every bootstrap having specimens (≥80%) of the TCGA-KIRC dataset. Cluster number *k* was between 2 and 10, and the optimum *k* was identified in accordance with the cumulative distribution function (CDF) and AUC.

### 2.3. Cancer Stem Cell

The expression data of pluripotent stem cell samples (embryonic stem cell (ESC) and induced pluripotent stem cell (iPSC)) from progenitor cell biology consortium (PCBC) database were used to predict and calculate the stem cell index by one-class logistic regression (OCLR) method. Firstly, only the sample data of ESC and iPSC are kept, which are collectively referred to as SC samples. The Ensembl IDs of SC samples are converted into gene symbol, and only the genes encoding proteins are kept. Finally, there are 78 SC samples, and the expression profiles of 8087 mRNA genes in each sample are kept. For the obtained expression profile, the average value was used to centralize each sample. Finally, the OCLR method in R package GelNet (V1.2.1) was used to calculate the weight vector of each gene on the processed data.

### 2.4. Weighted Correlation Network Analysis (WGCNA)

TCGA dataset was used for the identification of gene modules related to molecular subtypes by means of the R software package WGCNA [19]. Specifically, the samples were clustered first and then screened for coexpression modules. The coexpression network complies with the scale-free network when the logarithm log (*k*) of the node with the connection degree of *K* is negatively associated with the logarithm log (*P*(*k*)) of the probability of its occurrence, and the correlation coefficient is bigger than 0.85. Furthermore, an adjacency matrix was created by changing the gene expression similarity matrix. *β* is a soft thresholding parameter representing the Pearson's correlation coefficient for each gene pair [20]. We clustered genes using the average-linkage level clustering approach and a minimum of 200 genes per gene network module based on the topological overlap measure (TOM). After using the dynamic clipping approach to identify the gene modules, we examined the eigengenes of each module before doing cluster analysis on the modules. With the help of the parameters height = 0.25, deepSplit = 2, and minModuleSize = 200, the modules that are closer to one another are integrated to create new modules. The grey module includes a set of genes that cannot be integrated with other modules.

### 2.5. Mutation Analysis

A waterfall plot was generated to explore the detailed single-nucleotide variant (SNV) characteristics between molecular subtypes by using the “mutect2” [21] function in R software.

### 2.6. Cell-Type Identification by Estimating Relative Subsets of RNA Transcripts (CIBERSORT)

CIBERSORT analyses were utilized for comparing diversities in different immunocytes in molecular subtypes, and Wilcox test analyses were completed to identify the difference in 22 kinds of infiltrating immunocyte scores across these subtypes. The “ggplot2” package [22] was used to visualize the distributional status of the diversities in 22 kinds of infiltration immunocytes.

### 2.7. Computation of Immune Score, Estimate Score, and Stromal Score

The stromal level (StromalScore), immunocyte infiltration (ImmuneScore), and combination (ESTIMATEScore) of patients in the TCGA-KIRC cohort were calculated using R software Estimation of STromal and Immune cells in MAlignant Tumours using Expression data (ESTIMATE) arithmetic [20] and Wilcox test analysis to distinguish between molecular subtypes.

### 2.8. Tumor Immune Dysfunction and Exclusion (TIDE)

TIDE [23, 24], a calculation framework designed to assess the potential of cancer immune escape from the genetic expression profiles of tumor specimens, was used for predicting sample responses in the TCGA-KIRC datasets and comparing the proportion of treatment responses in different subtypes as well as the TIDE scores.

### 2.9. Drug Sensitivity Analysis

pRRophetic [25] was used to predict the sensitivity of sunitinib, cyclopamine, imatinib, crizotinib, erlotinib, sorafenib, dasatinib, and saracatinib to IC50 in molecular subtypes.

### 2.10. Statistical Analyses

All statistical analyses were performed using the R software (v3.6.3). The correlation matrices were conducted using Pearson or Spearman correlation. Wilcoxon test was conducted for the comparisons between the two groups. Survival differences were compared using KM curves with a log-rank test. *p* value < 0.05 was considered statistically significant.

## 3. Results

### 3.1. Three Necroptosis-Related Molecular Subtypes Were Screened

As described in Cluster Analysis in Material and Methods, 1301 and 4860 necroptosis score positively related genes (PCG) in TCGA-KIRC (*N* = 526) and RECA-EU (*N* = 91) datasets were determined, respectively. Subsequently, the intersection between them was also determined to obtain 914 PCG. Furthermore, univariate Cox survival analysis extracted 317 and 204 PCG related to KIRC prognosis in TCGA-KIRC and RECA-EU datasets, respectively, and by performing intersection analysis, 66 PCG were obtained (Figure S1).

**Figure 1:**
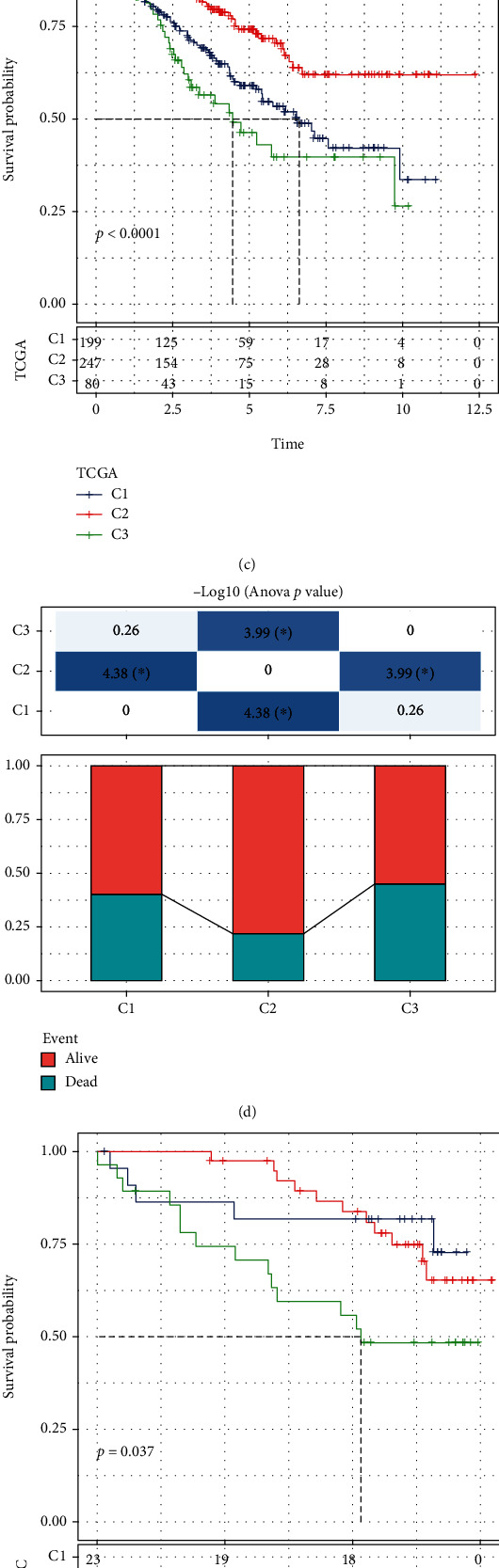
Three necroptosis-related molecular subtypes were screened. (a) Cumulative distribution function (CDF) curve and CDF Delta area in the TCGA-KIRC dataset. (b) Heat map of clusters when consensus *K* = 3. (c) Kaplan–Meier survival curve analysis among 3 clusters in TCGA-KIRC dataset. (d) Survival status of patients in 3 clusters in TCGA-KIRC dataset. (e) Kaplan–Meier survival curve analysis among 3 clusters in RECA-EU dataset. (f) Survival status of patients in 3 clusters in RECA-EU dataset.

Based on 66 PCG, patients in the TCGA-KIRC dataset were classified using ConsensusClusterPlus. When *K* = 3, the relative alteration in the area under the CDF curve was maximum (Figure 1(a)). Consequently, three subtypes, named, C1, C2, and C3, were screened (Figure 1(b)). Among them, samples in C2 had the best survival time, while the prognosis in patients in C3 was the worst (Figures 1(c) and 1(d)). The similar situation was observed in the RECA-EU dataset (Figures 1(e) and 1(f)). Of the distributional status of three clusters in diverse clinical characteristics (Figures S2A–S2G), remarkable diversity in age (Figure S2A), grade (Figure S2C), T stage (Figure S2D), M stage (Figure S2F), and stage (Figure S2G) in TCGA-KIRC cohort study was observed, while in the RECA-EU dataset, only age presented differences (Figure S3). In addition, we calculated the mRNAsi in TCGA and ICGA dataset and found that C3 had higher mRNAsi (Figure S4).

### 3.2. Genomic Mutation Characteristic Analysis

**Figure 2:**
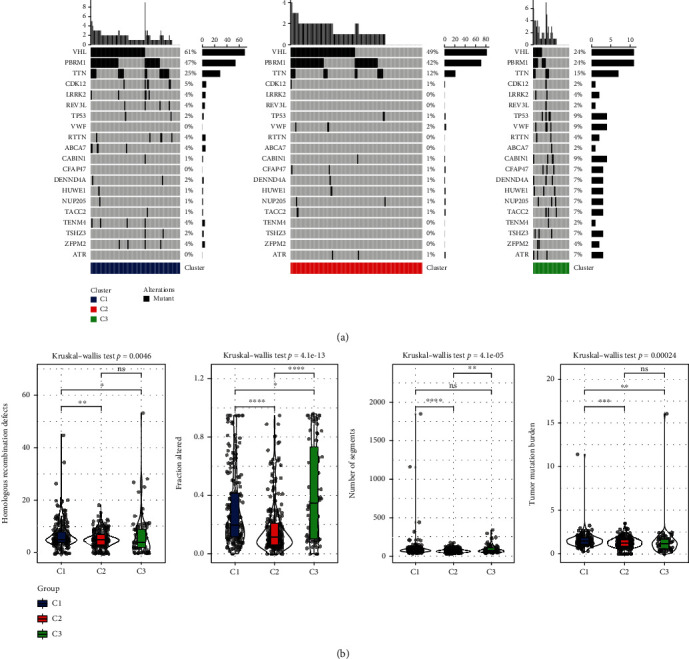
Genomic mutation characteristics among molecular subtypes. (a) Top 20 mutation genes in three subtypes. (b) The distribution of homologous recombination defects, fraction altered, number of segments, and tumor mutation burden among three subtypes. ns means no significance, ^∗^ means <0.05, ^∗∗^ means <0.01, ^∗∗∗^ means <0.001, and ^∗∗∗∗^ means <0.0001.

In TCGA dataset, we conducted an analysis of significantly mutant genes for KIRC samples across three molecular subtypes. The mutational landscapes of these three subgroups (Figure 2(a)) exhibited a distinct mutation ratio in VHL, PBPM1, and TTN. Besides, we also explored the distribution of fraction altered, tumor mutation burden, number of segments, and homologous recombination defects and presented differences among the three subtypes (Figure 2(b)).

### 3.3. Immune Infiltration Level Analysis among Subtypes

**Figure 3:**
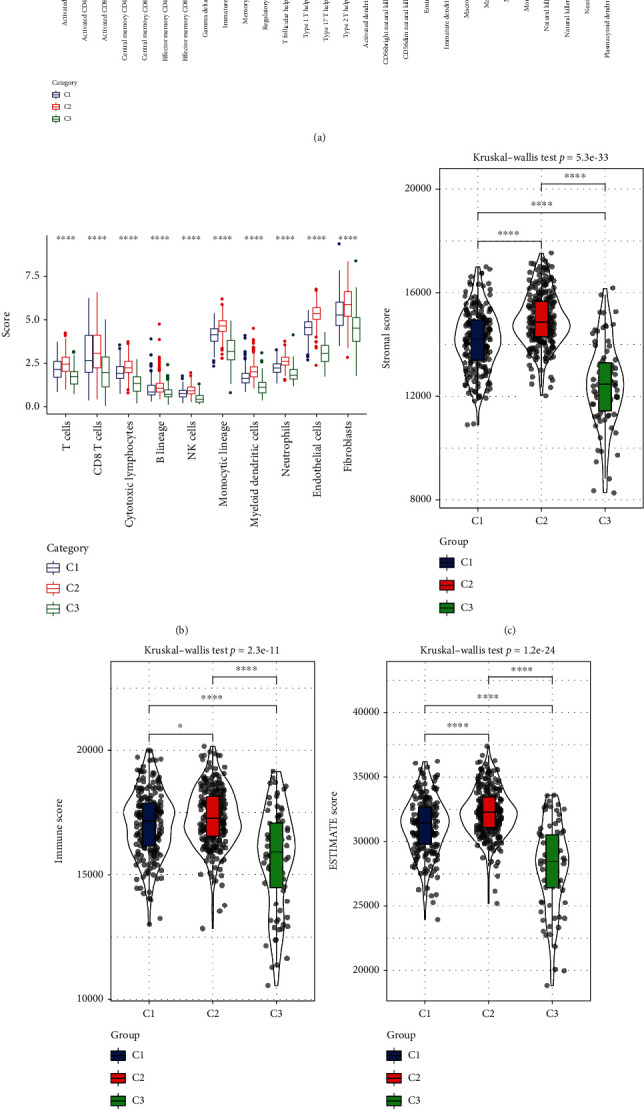
Immune infiltration status among molecular subtypes. (a) The differences of 28 immune cells' scores in three subtypes. (b) The differences of 10 immune cells' scores in three subtypes. (c) The differences of StromalScore in three subtypes. (d) The differences of ImmuneScore in three subtypes. (e) The differences of ESTIMATEScore in three subtypes. ns means no significance, ^∗^ means <0.05, ^∗∗^ means <0.01, ^∗∗∗^ means <0.001, and ^∗∗∗∗^ means <0.0001.

We hypothesized that differential immunological enrichment may be reflected in subtypes. First, using the TCGA-KIRC dataset, a histogram was created using ssGSEA to display the relative abundance of 28 immune infiltrating cell subpopulations. We observed 27 kinds of immunocytes with significantly different distributions among three subtypes. The most enriched is in C2, such as activated, effector memory CD4+/CD8+ T cells, CD4+/CD8+ T cells, and natural killer T cells (Figure 3(a)). Afterward, our team evaluated the 10 kinds of immune cells' scores using microenvironment cell population-counter (MCP-counter) methods, and all high enriched in C2 (Figure 3(b)). Everything goes well, C2 sufferers had a higher score for StromalScore, ImmuneScore, and ESTIMATEScore (Figures 3(c)–3(e)).

### 3.4. Immunotherapy/Chemotherapy Analysis among Subtypes

**Figure 4:**
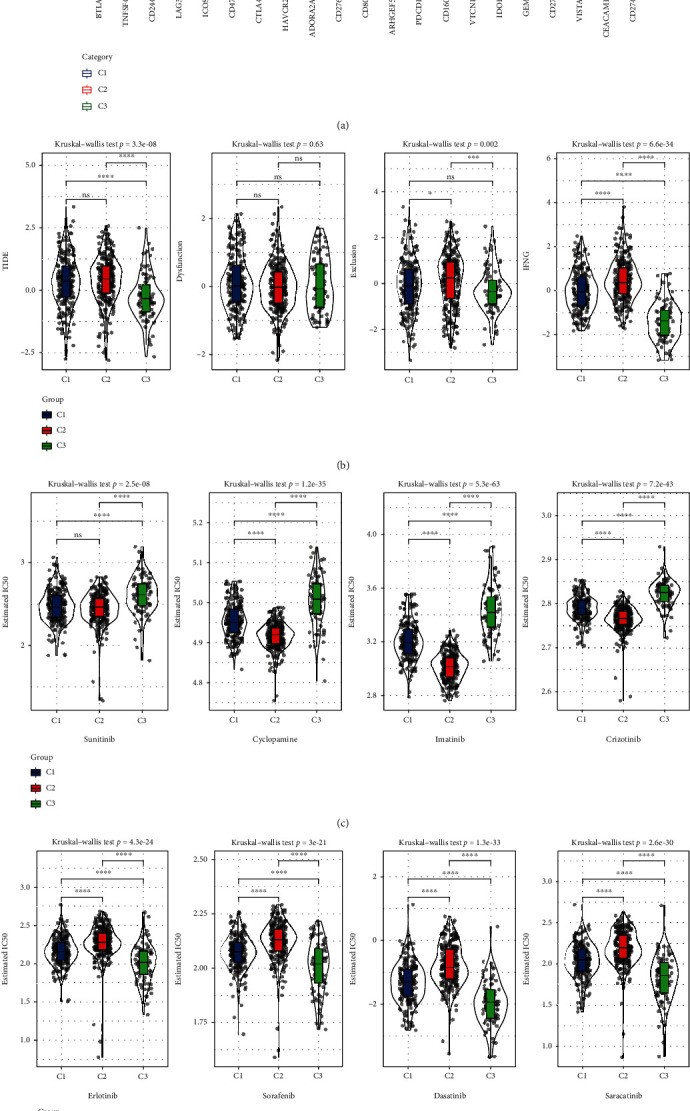
Immunotherapy/chemotherapy analysis among subtypes. (a) The expression differences of 21 immune checkpoint inhibitors (ICI) among three subtypes. (b) TIDE analysis of the immunotherapy response differences among three subtypes. (c) Patients in C3 were more sensitive to sunitinib, cyclopamine, imatinib, and crizotinib. (c) Samples in C2 were more sensitive to erlotinib, sorafenib, dasatinib, and saracatinib. ns means no significance, ^∗^ means <0.05, ^∗∗^ means <0.01, ^∗∗∗^ means <0.001, and ^∗∗∗∗^ means <0.0001.

Immune checkpoint inhibitor (ICI) therapies represented by anti-PD-1/L1 agents have undoubtedly made a great breakthrough in antitumor therapy. Therefore, 21 ICI were acquired from the HisgAtlas database, and all had high expression in C2 than those in C1/C3 (Figure 4(a)). Moreover, the estimated scores of immune therapy biomarkers were computed with the help of TIDE arithmetic. Our team assessed the qualities of TIDE, IFNG, and T cell exclusion (exclusion), which were greater in the C2 group versus the C1/C3 group except for T cell function disorder scores (dysfunctions) (Figure 4(b)).

We also investigate how each of these 3 groups responded to various commonly used chemotherapy drugs. The findings showed that drugs including sunitinib, cyclopamine, imatinib, and crizotinib had higher IC50 values in C3, indicating that individuals in C3 were significantly more susceptible to such drugs (Figure 4(c)).

On the other hand, drugs including erlotinib, sorafenib, dasatinib, and saracatinib had high IC50 values in C2, indicating remarkably increased sensitivity of C2 to them (Figure 4(d)).

### 3.5. Coexpression Network of Subtypes Using WGCNA

**Figure 5:**
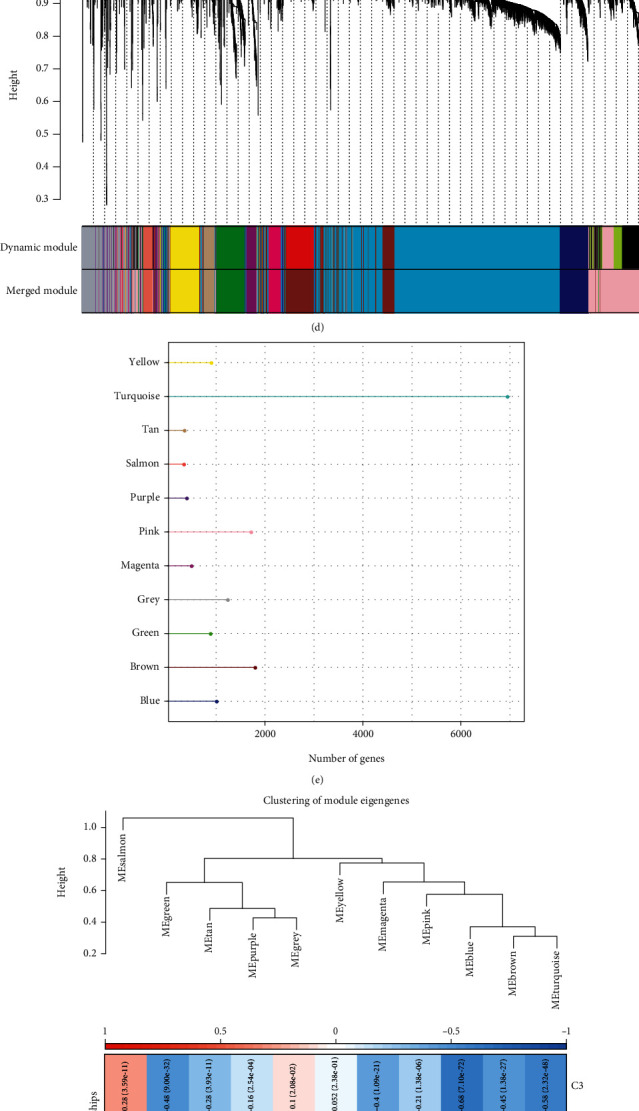
WGCNA. (a) Clustering tree of samples in TCGA-KIRC dataset. (b) An examination of the scale-free fit index for different soft-thresholding powers (*β*). (c) Assessment of the mean connectivity for different soft-thresholding powers. (d) Dendrogram displaying all differentially expressed genes, clustered based on a dissimilarity metric (1-TOM). (e) Number of genes in 11 modules. (f) The correlation analysis between three subtypes and 11 modules. (g) The correlation analysis between gene significance in C2 and blue module membership.

A dendrogram of samples (TCGA-KIRC) with clinical features was created using the average linkage method and Pearson's correlation approach (Figure 5(a)). The soft threshold power (*β*) of 8 in the TCGA-KIRC dataset was estimated to make sure of a scale-free network (Figures 5(b) and 5(c)). Hierarchical clustering helped to identify 11 modules (Figure 5(d)). Additionally, the number of genes in each of the 11 modules was computed, with the turquoise module having the largest number of genes (Figure 5(e)). The correlation analysis between molecular subtypes and 11 modules showed that the blue module was negatively correlated with C3, while positively correlated with C2 (Figure 5(f)). Moreover, module membership in blue was highly positively correlated with gene significance for C2 (Figure 5(g)). To determine the importance of the blue module, ClusterProfiler in the R package was applied for function enrichment in the blue module. The findings demonstrated that the blue module was enriched in pathways that are linked to tumors, including the Rap1 signaling pathway, the PI3K-Akt signaling pathway, and the Notch signaling pathway (Figure S4). Thus, the blue module was considered the hub gene module associated with the molecular subtype.

### 3.6. Identification of Necroptosis-Related Signature

**Figure 6:**
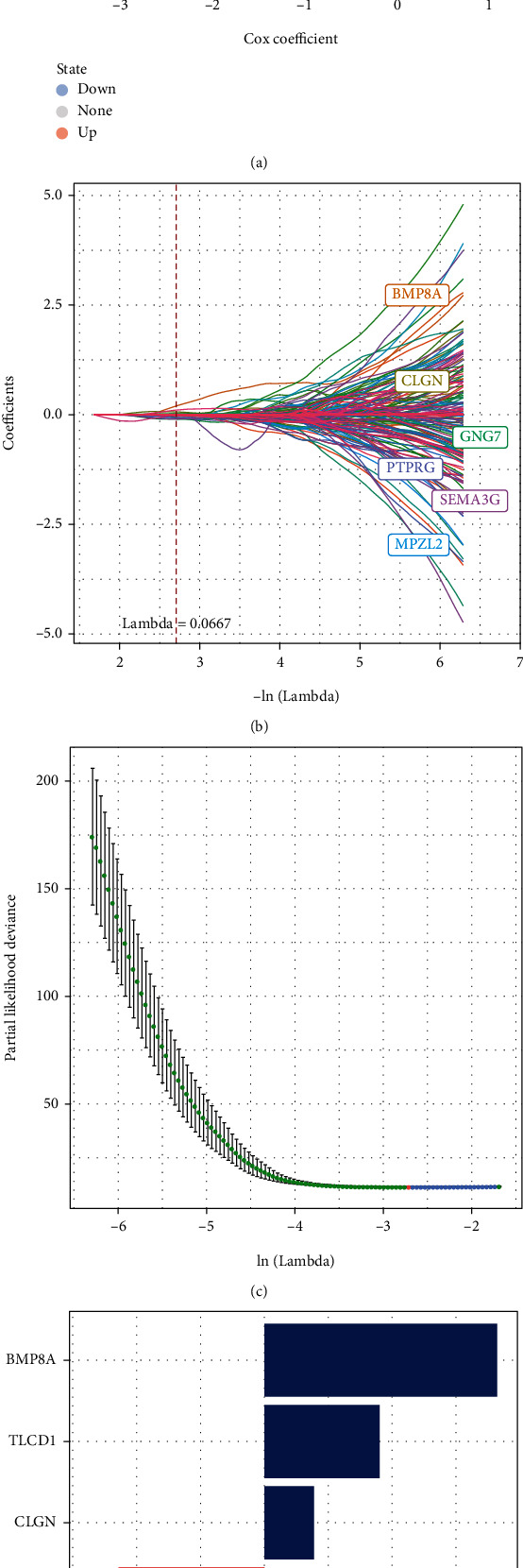
Identification of promising candidates. (a) Through the survival analysis of the genes in the blue module, a total of viable candidates were found. (b) The coefficients of various promising candidates. (c) A confidence interval under lambda. (d) Five promising genes were identified.

Using univariate Cox survival analysis, the TCGA-KIRC training dataset was examined for 13 upregulated genes and 565 downregulated genes related to the prognosis of patients with KIRC (Figure 6(a)). LASSO regression was executed while fitting the generalized linear model according to the variable selection and regularization characteristics in order to identify hub genes for calculating the prognosis of high-performance patients (Figures 6(b) and 6(c)), and finally, we identified 5 hub necroptosis genes (BMP8A, TLCD1, CLGN, GDF7, and RARB) (Figure 6(d)). The formula was accordingly constructed as below: RiskScore = (0.729^∗^expression level of BMP8A) + (0.361^∗^ expression level of TLCD1) + (0.156^∗^ expression level of CLGN) − (0.457^∗^ expression level of GDF7) − (0.546^∗^ expression level of RARB).

### 3.7. Prognostic Performance Test of Necroptosis-Related Signature

**Figure 7:**
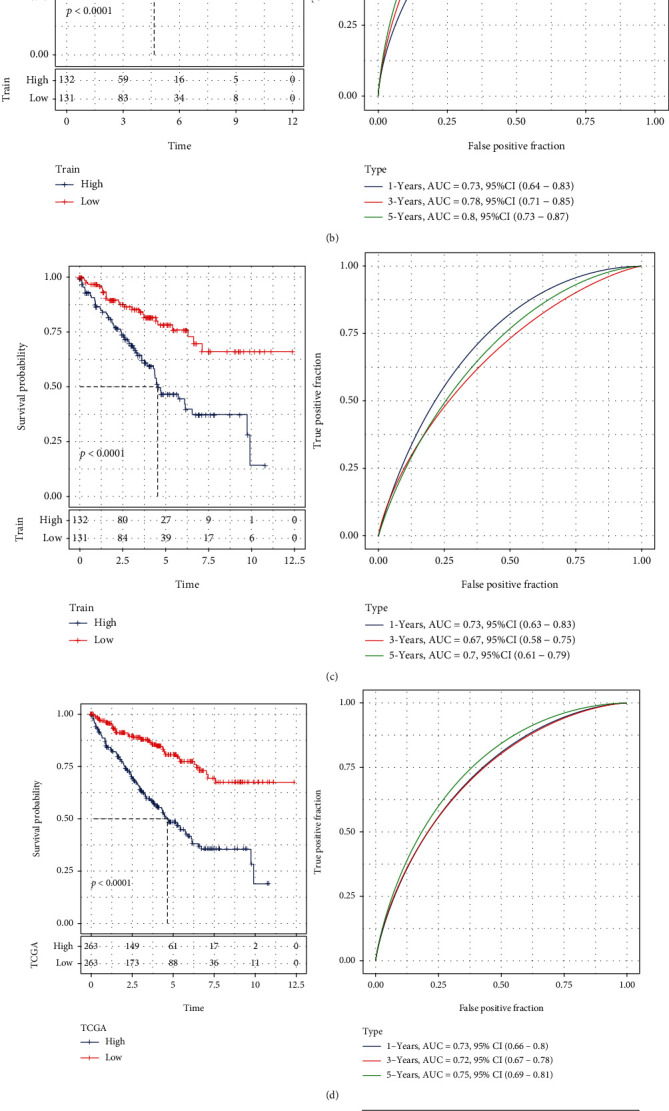
Prognostic ability of necroptosis-related gene signature. (a) A forest map presented 5 necroptosis-related gene signature derived by Cox proportional hazard regression in the TCGA-KIRC cohort. (b) KM survival and ROC of 5 necroptosis-related gene signature in TCGA-KIRC training cohort. (c) KM survival and ROC of 5 necroptosis-related gene signature in TCGA-KIRC test cohort. (d) KM survival and ROC of 5 necroptosis-related gene signature in the entire TCGA-KIRC cohort. (e) KM survival and ROC of 5 necroptosis-related gene signature in RECA-EU cohort.

The risk assessment of prognosis-related genes was achieved through univariate Cox analysis. In KIRC patients, BMP8A, GLGN, and TLCD1 were identified as risk genes for the prognosis, whereas RARB and GDF7 were recognized as protective genes for KIRC patients (Figure 7(a)).

**Figure 8:**
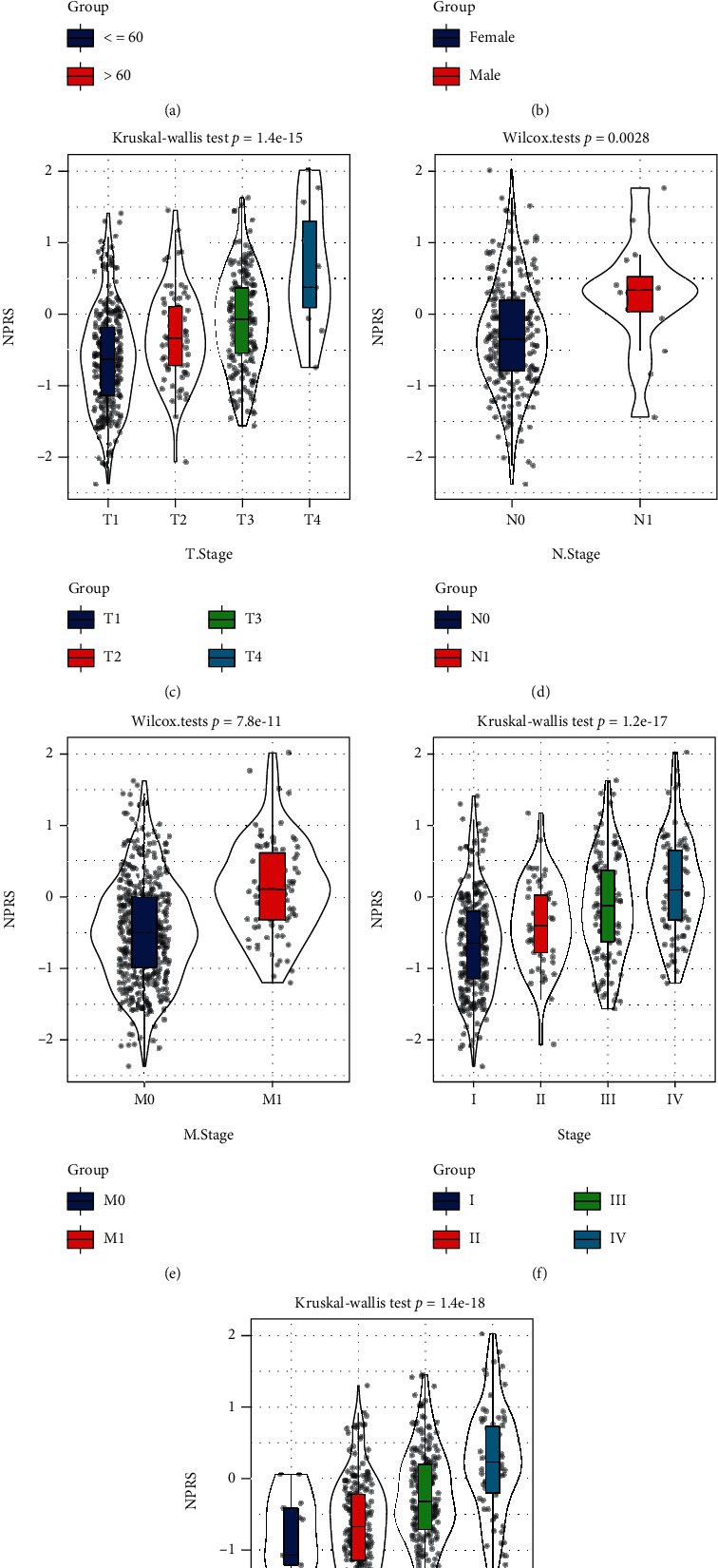
The distribution of RiskScore in (a) age, (b) gender, (c) grade, (d) T stage, (e) N stage, (f) M stage, and (g) stage.

Next, the RiskScore of patients in the TCGA-KIRC training dataset was analyzed in accordance with the above formula. Two groups were created, namely, the high-RiskScore group and the low-RiskScore group, for dividing the patients. According to the KM survival curve, in the TCGA-KIRC training dataset, the low RS group performed better in OS than the high RS group. In the TCGA-KIRC training group, the AUCs for 1-, 3-, and 5-year survival were 0.73, 0.78, and 0.8, correspondingly (Figure 7(b)). In the TCGA-KIRC test cohort, samples in the low group had a longer survival time than those in the high group, and the AUCs for 1-, 3-, and 5-year survival in the test cohort were 0.73, 0.67, and 0.7, correspondingly (Figure 7(c)). Overall, in the TCGA-KIRC cohort, samples in the low group generally had better survival time than those in the high group; the AUCs for 1-, 3-, and 5-year survival were, 0.73, 0.72, and 0.75, correspondingly (Figure 7(d)). Similar results were seen in the RECA-EU dataset, where samples in the low group had a longer survival time than the high group, with AUCs for 1-, 3-, and 5-year survival in the RECA-EU cohort being 0.77, 0.68, and 0.71, correspondingly (Figure 7(e)). Of the distributional status of two groups in diverse clinical characteristics (Figures 8(a)–8(g)), remarkable diversity in T stage (Figure 8(c)), N stage (Figure 8(d)), M stage (Figure 8(e)), stage (Figure 8(f)), and grade (Figure 8(g)) in TCGA-KIRC cohort study was observed.

### 3.8. Genomic Mutation Characteristic Analysis

**Figure 9:**
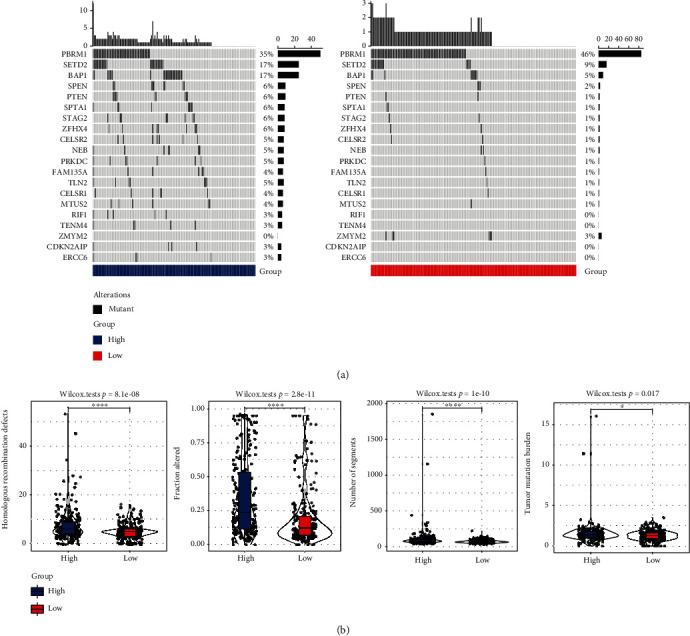
Genomic mutation characteristics in high group and low group. (a) Top 20 mutation genes in the high group and the low group. (b) The distribution of homologous recombination defects, fraction altered, number of segments, and tumor mutation burden between the high group and the low group. ^∗^ means <0.05 and ^∗∗∗∗^ means <0.0001.

The high group and the low group in the TCGA-KIRC cohort were subjected to an analysis of significantly mutated genes for KIRC samples, and the mutational profiles of these genes in the two groups (Figure 9(a)) demonstrated a distinct mutation ratio in PBPM1, SETD2, and BAP1. On the other hand, we also explored the distribution of fraction altered, number of segments, tumor mutation burden, and homologous recombination defects. The findings revealed that the high group had higher values than the low group (Figure 9(b)).

### 3.9. Pathways Were Inhibited in the High Group

The gene set enrichment analysis (GSEA) was employed to investigate the difference between high and low groups as well as their involved pathways and functions. The RiskScore was used as the reference phenotype. The GSEA revealed that tumor-associated pathways such as KEGG_P53_SIGNALING_PATHWAY, KEGG_BASE_EXCISION_REPAIR, and KEGG_HOMOLOGOUS_RECOMBINATION were positively correlated with RiskScore (Figure S5A). In the TCGA-KIRC cohort and RECA-EU dataset, respectively, 14 pathways and 13 pathways were inhibited in the high group compared to the low group (Figure S5B).

### 3.10. RiskScore and Clinical Pathology Characters Synergistically Predicted the Survival Probability of KIRC Patients

**Figure 10:**
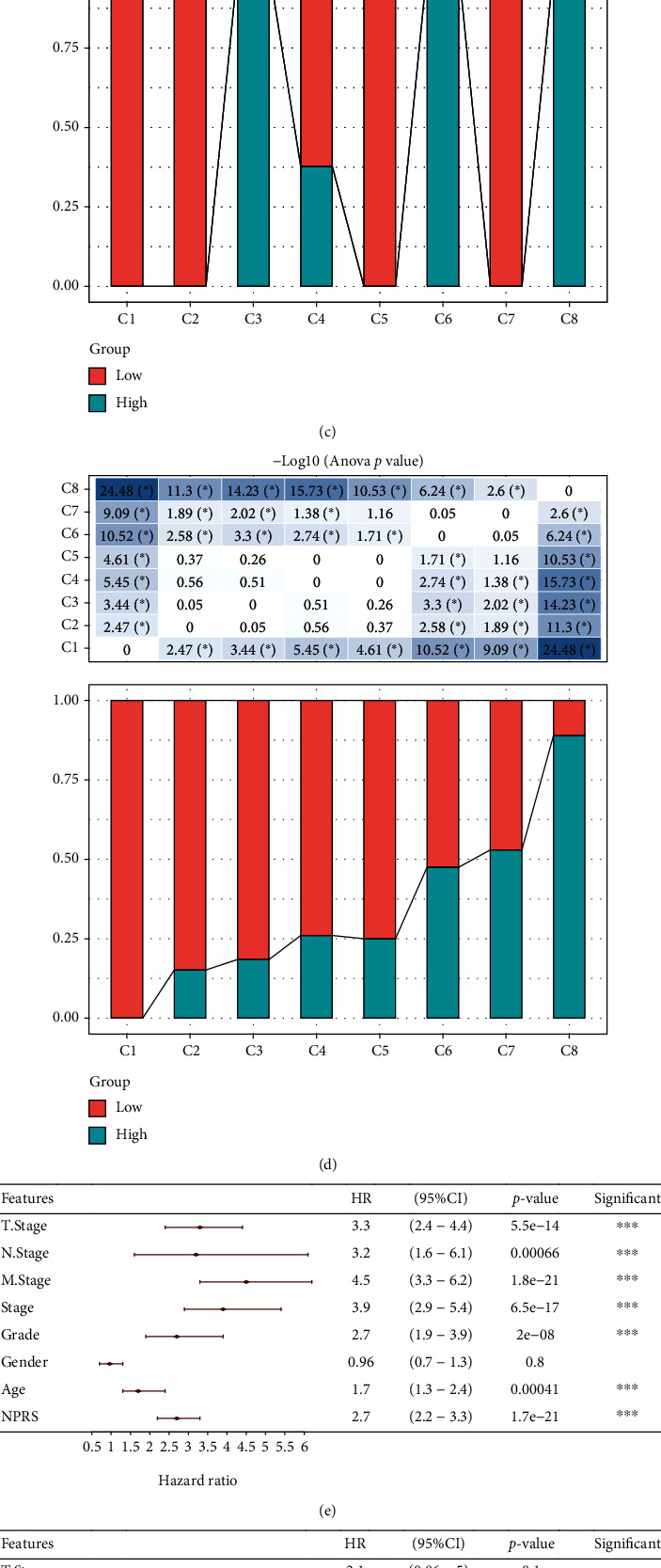
RiskScore and clinical pathology characters synergistically predicted the survival probability of KIRC patients. (a) Eight different risk subgroups were identified. (b) The overall survival among 8 subgroups had significance. (c) Patients in risk subgroups C3, C6, and C8 belong to the high group, while patients in risk subgroups C1, C2, C5, and C7 belong to the low group. (d) The survival status in 8 subgroups had significant differences. (e, f) M stage, age, and RiskScore were independent prognostic indicators, according to univariate and multivariate Cox survival analysis. (g) A prognostic nomogram to anticipate the 1-, 3-, and 5-year OS of KIRC patients using the M stage, age, and RiskScore. (h) The accuracy and robustness of the prognostic nomogram were demonstrated by the calibration curve. (i) The outcome of decision curve analysis (DCA) demonstrated that among clinical factors used in clinical decision-making, the RiskScore acted as the most exact prognostic indicator.

The age, gender, N stage, M stage, T stage, grade, and RiskScore in the TCGA-KIRC cohort were taken into account when constructing the decision tree, and the results indicated that RiskScore, stage, age, grade, and M stage were left in the decision tree. As a result, 8 different risk subgroups were identified (Figure 10(a)) and the overall survival among them showed significance (Figure 10(b)). Among them, patients in risk subgroups C3, C6, and C8 belong to the high group, while patients in risk subgroups C1, C2, C5, and C7 belong to the low group (Figure 10(c)). In addition, the survival status in 8 subgroups had significant differences (Figure 10(d)). The age, M stage, and RiskScore were independent prognostic variables, according to univariate and multivariate Cox survival analysis results (Figures 10(e) and 10(f)). A prognostic nomogram based on M stage, age, and RiskScore was integrated together to compute 1-, 3-, and 5-year OS of KIRC patients for the purpose of providing a quantitative method for anticipating the prognosis of these patients (Figure 10(g)). The prognostic nomogram's accuracy and robustness were established via the calibration curve (Figure 10(h)). The results of decision curve analysis (DCA) showed that, among the several clinical factors used in clinical decision-making, the RiskScore acted as the most reliable prognostic indicator (Figure 10(i)).

## 4. Discussion

In this study, 66 genes associated with necroptosis were analyzed in the TCGA-KIRC dataset and RECA-EU dataset, and 66 PCGs were used to identify three molecular subtypes. Patients in C2 had better survival time, enhanced immune status, and weak benefits from immunotherapy. Moreover, 5 necroptosis-related prognostic gene-based signature was built to anticipate the predicted prognosis of KIRC patients. Patients in the low group had a worse prognosis, and the nomogram with the known risk factors and RiskScore had a better prognosis effect.

As we all know, necrosis is an alternative to programmed cell death that can deal with apoptosis resistance and stimulate and boost antitumor immunity in tumor therapy [26]. Necrosis may act as a tumor suppressor, making it a potentially useful cancer treatment. Numerous necroptosis-associated gene-based signatures have been proposed to have significant roles in a variety of malignancies to date. In order to predict the outcome of pancreatic cancer, Wu et al. reported a signature incorporating 25 genes linked to necroptosis [25]. Wang et al. presented a thorough bioinformatics analysis and proposed a necroptosis-related prognostic signature in stomach adenocarcinoma [27]. The prognosis of patients with pancreatic cancer was successfully determined using a unique five necroptosis-related gene signature [28]. Additionally, in renal carcinoma, some research about necroptosis was reported to predict patients' prognosis, immune microenvironment, and immunotherapy [17, 29, 30]. The findings presented above demonstrated that necroptosis had a significant role in regulating the growth of tumors. As a result, we discovered two molecular subtypes that have different prognoses as well as 66 necroptosis-related genes linked to KIRC prognosis. Furthermore, 5 necroptosis-related gene signatures were determined to anticipate the prognosis of KIRC.

Among the 5 necroptosis-related genes included in the prognostic signature, patients with renal cell carcinomas had high levels of BMP8A expression, which promoted survival and drug resistance [31]. A study revealed that CLGN was strongly expressed in aldosterone-producing adenomas and aldosterone-producing cell clusters [32]. Rs3072 at GDF7 was associated with the progression of esophageal adenocarcinoma [33]. Some studies have suggested that a loss of RARB expression indicated progressive behavior in premalignant and malignant tissues, as well as the immortal cells [34–36]. Furthermore, we also established a nomogram for estimating the overall survival (OS) of KIRC patients in combination with RiskScore and clinicopathological characteristics.

We are obligated to acknowledge the limitations of this study even though we have used bioinformatics methods on a large sample for the identification of genetic subgroups of KIRC with significant prognostic differences. We intend to emphasize fundamentally experimental and functionally in-depth research more in the future. Other considerations were not taken into account on our end because the samples lacked essential data on clinical follow-up, most notably diagnostic specifics such as whether or not the patients had other health conditions when differentiating the molecular subtypes.

## 5. Conclusions

In conclusion, three subgroups were created based on genes associated with necroptosis in order to guide tailored therapy for KIRC patients and build 5 necroptosis-related gene signature for predicting OS. Collectively, we provided strong preclinical evidence that necroptosis-related subtypes and RiskScore may be effective for the precise treatment of KIRC patients.

## Data Availability

The data analyzed in this study are available upon reasonable request.

## Supplementary Materials

Supplementary Materials Figure S1: intersection analysis between TCGA-KIRC dataset and RECA-EU dataset to acquire necroptosis score positively related genes. Figure S2: the distribution of age (A), gender (B), grade (C), T stage (D), N stage (E), M stage (F), and stage (G) in three subtypes in the TCGA-KIRC dataset. Figure S3: the distribution of age (A) and gender (B) in three subtypes in RECA-EU dataset. Figure S4: function enrichment analysis. A: BP of GO analysis. B: CC of GO analysis. C: MF of GO analysis. D: KEGG analysis. Figure S5: functional enrichment analysis. A: the GSEA revealed that tumor-associated pathways were correlated with RiskScore. B: 14 pathways and 13 pathways were inhibited in the high group than in the low group in the TCGA-KIRC cohort and RECA-EU dataset, respectively.

## References

[1] Escudier B., Porta C., Schmidinger M. (2019). Renal cell carcinoma: ESMO clinical practice guidelines for diagnosis, treatment and follow-up. *Annals of Oncology*.

[2] Siegel R. L., Miller K. D., Fuchs H. E., Jemal A. (2022). Cancer statistics, 2022. *CA: a Cancer Journal for Clinicians*.

[3] Hsieh J. J., Purdue M. P., Signoretti S. (2017). Renal cell carcinoma. *Nature Reviews Disease Primers*.

[4] He S., Wang L., Miao L. (2009). Receptor interacting protein kinase-3 determines cellular necrotic response to TNF-*α*. *Cell*.

[5] Petrie E. J., Czabotar P. E., Murphy J. M. (2019). The structural basis of necroptotic cell death signaling. *Trends in Biochemical Sciences*.

[6] Yang F., Shang L., Wang S. (2019). TNF*α*-mediated necroptosis aggravates ischemia-reperfusion injury in the fatty liver by regulating the inflammatory response. *Oxidative Medicine and Cellular Longevity*.

[7] Pasparakis M., Vandenabeele P. (2015). Necroptosis and its role in inflammation. *Nature*.

[8] Zhou K., Shi L., Wang Z. (2017). RIP1-RIP3-DRP1 pathway regulates NLRP3 inflammasome activation following subarachnoid hemorrhage. *Experimental Neurology*.

[9] Koo G. B., Morgan M. J., Lee D. G. (2015). Methylation-dependent loss of RIP3 expression in cancer represses programmed necrosis in response to chemotherapeutics. *Cell Research*.

[10] Geserick P., Wang J., Schilling R. (2015). Absence of RIPK3 predicts necroptosis resistance in malignant melanoma. *Cell Death & Disease*.

[11] Feng X., Song Q., Yu A., Tang H., Peng Z., Wang X. (2015). Receptor-interacting protein kinase 3 is a predictor of survival and plays a tumor suppressive role in colorectal cancer. *Neoplasma*.

[12] Bozec D., Iuga A. C., Roda G., Dahan S., Yeretssian G. (2016). Critical function of the necroptosis adaptor RIPK3 in protecting from intestinal tumorigenesis. *Oncotarget*.

[13] McCormick K. D., Ghosh A., Trivedi S. (2016). Innate immune signaling through differential RIPK1 expression promote tumor progression in head and neck squamous cell carcinoma. *Carcinogenesis*.

[14] Thorsson V., Gibbs D. L., Brown S. D. (2018). The immune landscape of cancer. *Immunity*.

[15] Setton J., Reis-Filho J. S., Powell S. N. (2021). Homologous recombination deficiency: how genomic signatures are generated. *Current Opinion in Genetics & Development*.

[16] Jardim D. L., Goodman A., de Melo Gagliato D., Kurzrock R. (2021). The challenges of tumor mutational burden as an immunotherapy biomarker. *Cancer Cell*.

[17] Xin S., Mao J., Duan C. (2022). Identification and quantification of necroptosis landscape on therapy and prognosis in kidney renal clear cell carcinoma. *Frontiers in Genetics*.

[18] Wilkerson M. D., Hayes D. N. (2010). ConsensusClusterPlus: a class discovery tool with confidence assessments and item tracking. *Bioinformatics*.

[19] Langfelder P., Horvath S. (2008). WGCNA: an R package for weighted correlation network analysis. *BMC Bioinformatics*.

[20] Horvath S., Zhang B., Carlson M. (2006). Analysis of oncogenic signaling networks in glioblastoma identifies ASPM as a molecular target. *Proceedings of the National Academy of Sciences of the United States of America*.

[21] Pei S., Liu T., Ren X., Li W., Chen C., Xie Z. (2021). Benchmarking variant callers in next-generation and third-generation sequencing analysis. *Briefings in Bioinformatics*.

[22] Ito K., Murphy D. (2013). Application of *ggplot2* to pharmacometric graphics. *CPT: Pharmacometrics & Systems Pharmacology*.

[23] Fu J., Li K., Zhang W. (2020). Large-scale public data reuse to model immunotherapy response and resistance. *Genome Medicine*.

[24] Jiang P., Gu S., Pan D. (2018). Signatures of T cell dysfunction and exclusion predict cancer immunotherapy response. *Nature Medicine*.

[25] Geeleher P., Cox N., Huang R. S. (2014). pRRophetic: an R package for prediction of clinical chemotherapeutic response from tumor gene expression levels. *PLoS One*.

[26] Linkermann A., Green D. R. (2014). Necroptosis. *The New England Journal of Medicine*.

[27] Wang N., Liu D. (2021). Identification and validation a necroptosis-related prognostic signature and associated regulatory axis in stomach adenocarcinoma. *Oncotargets and Therapy*.

[28] Shi H., Peng Q., Zhou X., He Y., Sun S. (2022). An efficient signature based on necroptosis-related genes for prognosis of patients with pancreatic cancer. *Frontiers in Genetics*.

[29] Chen W., Lin W., Wu L., Xu A., Liu C., Huang P. (2022). A novel prognostic predictor of immune microenvironment and therapeutic response in kidney renal clear cell carcinoma based on necroptosis-related gene signature. *International Journal of Medical Sciences*.

[30] Gu J., He Z., Huang Y. (2022). Clinicopathological and prognostic value of necroptosis-associated lncRNA model in patients with kidney renal clear cell carcinoma. *Disease Markers*.

[31] Yu Y. P., Cai L. C., Wang X. Y. (2020). BMP8A promotes survival and drug resistance via Nrf 2/TRIM24 signaling pathway in clear cell renal cell carcinoma. *Cancer Science*.

[32] Itcho K., Oki K., Gomez-Sanchez C. E. (2020). Endoplasmic reticulum chaperone calmegin is upregulated in aldosterone-producing adenoma and associates with aldosterone production. *Hypertension*.

[33] Becker J., May A., Gerges C. (2016). The Barrett-associated variants at GDF7 and TBX5 also increase esophageal adenocarcinoma risk. *Cancer Medicine*.

[34] Bouras E., Karakioulaki M., Bougioukas K. I., Aivaliotis M., Tzimagiorgis G., Chourdakis M. (2019). Gene promoter methylation and cancer: an umbrella review. *Gene*.

[35] Shan L., Liu W., Zhan Y. (2021). LncRNA HAND2-AS1 exerts anti-oncogenic effects on bladder cancer via restoration of RARB as a sponge of microRNA-146. *Cancer Cell International*.

[36] Osumi T., Tsujimoto S. I., Tamura M. (2018). Recurrent RARB translocations in acute promyelocytic leukemia lackingRARAtranslocation. *Cancer Research*.

