# Supplementary figures and images for: Identification and Validation of a Necroptosis-Related Prognostic Signature for Kidney Renal Clear Cell Carcinoma
Source: Stem Cells Int. 2023 Mar 3;2023:8446765. doi: 10.1155/2023/8446765 (PMC10005877; doi:10.1155/2023/8446765)

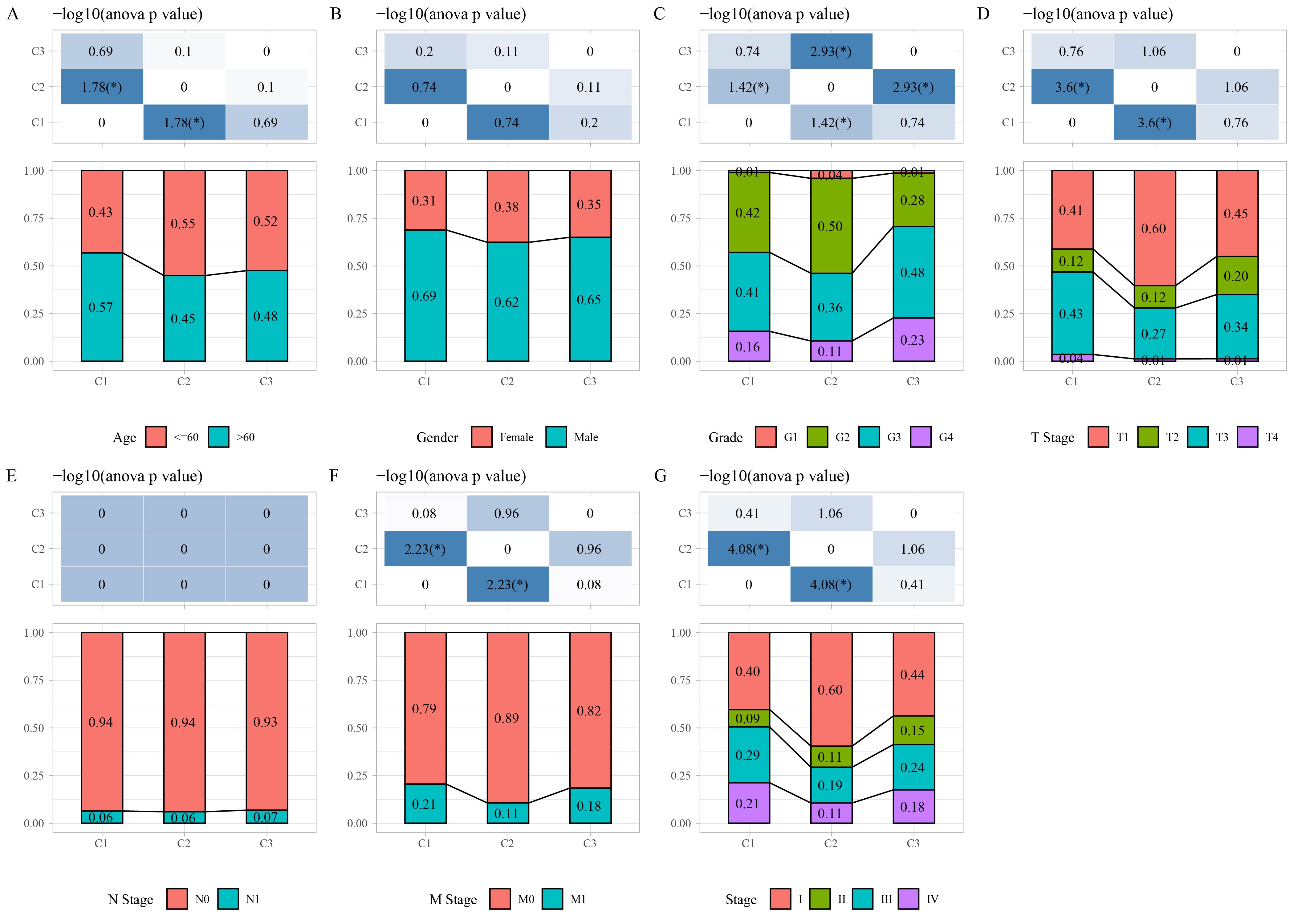

Supplement: Supplementary Materials — Figure S1: intersection analysis between TCGA-KIRC dataset and RECA-EU dataset to acquire necroptosis score positively related genes. Figure S2: the distribution of age (A), gender (B), grade (C), T stage (D), N stage (E), M stage (F), and stage (G) in three subtypes in the TCGA-KIRC dataset. Figure S3: the distribution of age (A) and gender (B) in three subtypes in RECA-EU dataset. Figure S4: function enrichment analysis. A: BP of GO analysis. B: CC of GO analysis. C: MF of GO analysis. D: KEGG analysis. Figure S5: functional enrichment analysis. A: the GSEA revealed that tumor-associated pathways were correlated with RiskScore. B: 14 pathways and 13 pathways were inhibited in the high group than in the low group in the TCGA-KIRC cohort and RECA-EU dataset, respectively. [file 8446765.f1.zip › Figrue S2.jpg]

ANOVA tests  $p=7e-28$ 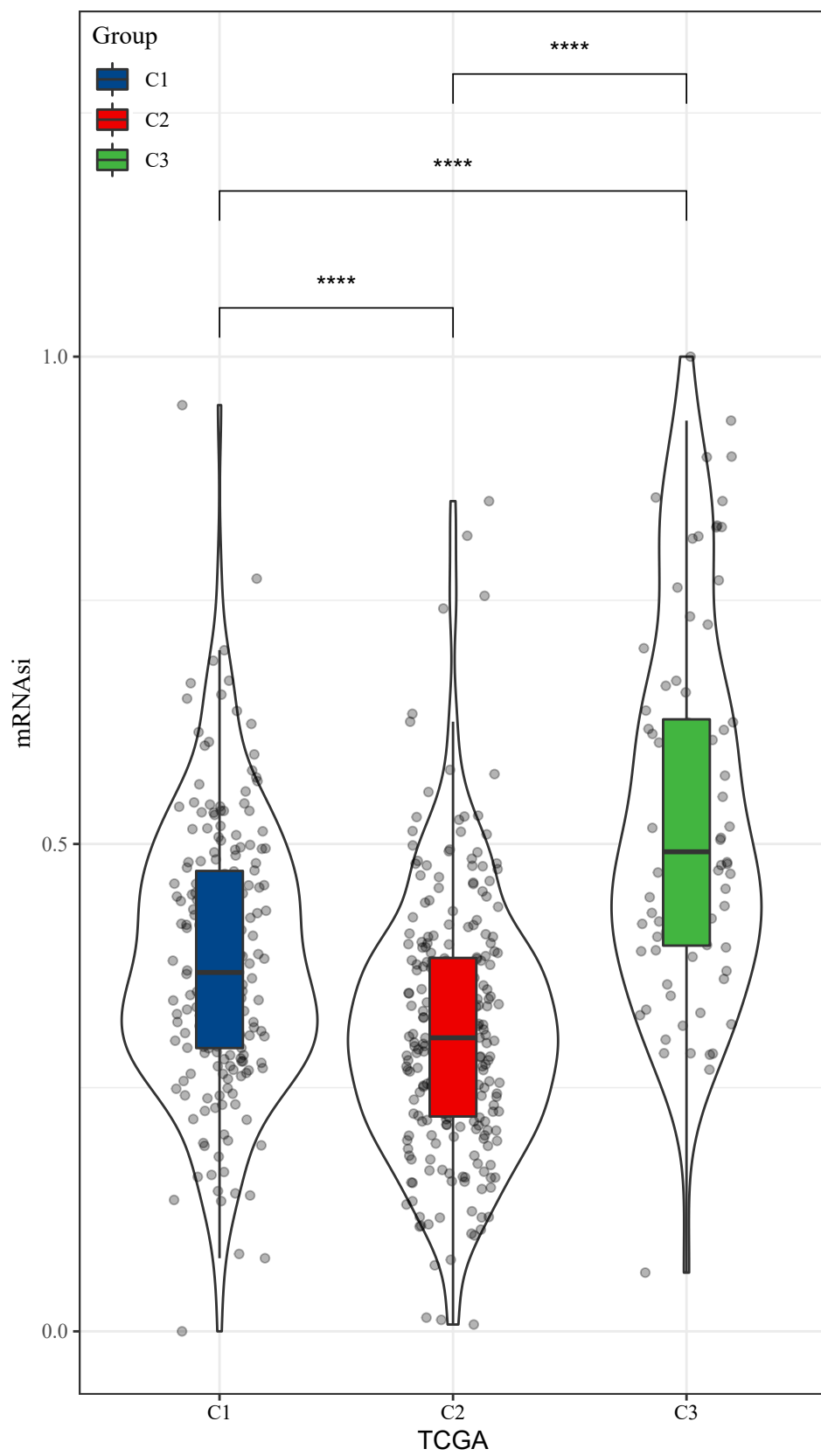ANOVA tests  $p=0.00031$ 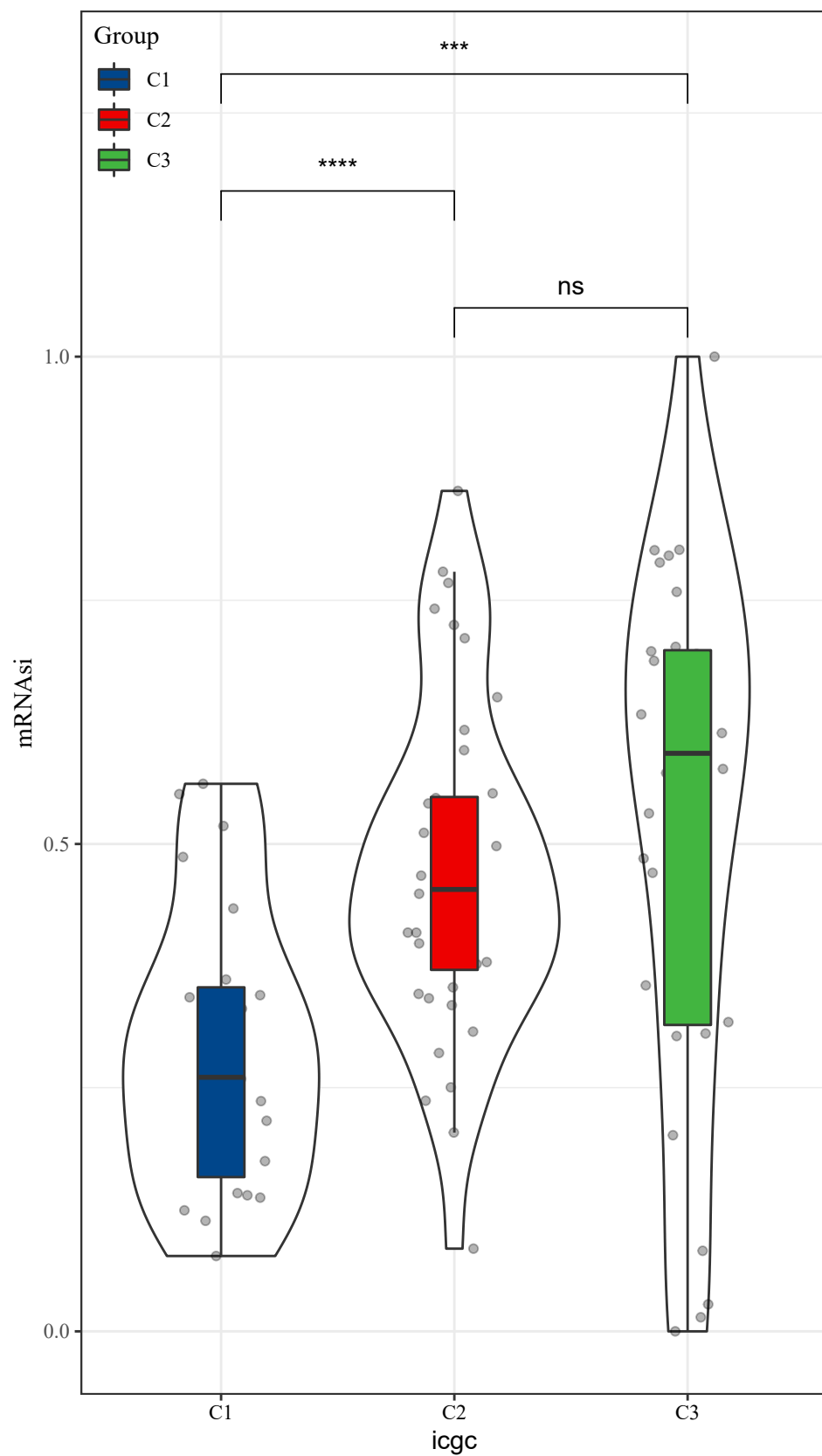

Supplement: Supplementary Materials — Figure S1: intersection analysis between TCGA-KIRC dataset and RECA-EU dataset to acquire necroptosis score positively related genes. Figure S2: the distribution of age (A), gender (B), grade (C), T stage (D), N stage (E), M stage (F), and stage (G) in three subtypes in the TCGA-KIRC dataset. Figure S3: the distribution of age (A) and gender (B) in three subtypes in RECA-EU dataset. Figure S4: function enrichment analysis. A: BP of GO analysis. B: CC of GO analysis. C: MF of GO analysis. D: KEGG analysis. Figure S5: functional enrichment analysis. A: the GSEA revealed that tumor-associated pathways were correlated with RiskScore. B: 14 pathways and 13 pathways were inhibited in the high group than in the low group in the TCGA-KIRC cohort and RECA-EU dataset, respectively. [file 8446765.f1.zip › Figrue S4.pdf]

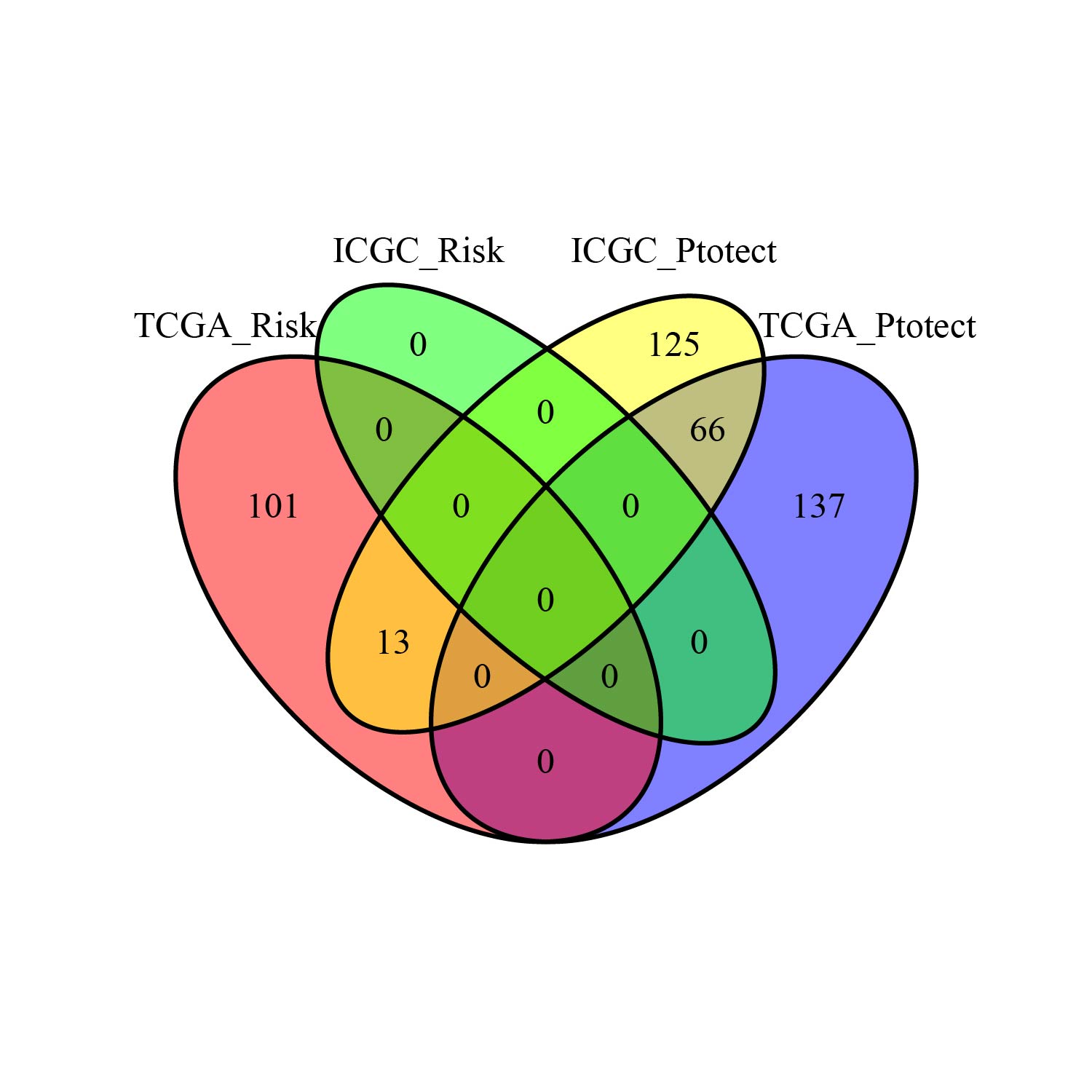

Supplement: Supplementary Materials — Figure S1: intersection analysis between TCGA-KIRC dataset and RECA-EU dataset to acquire necroptosis score positively related genes. Figure S2: the distribution of age (A), gender (B), grade (C), T stage (D), N stage (E), M stage (F), and stage (G) in three subtypes in the TCGA-KIRC dataset. Figure S3: the distribution of age (A) and gender (B) in three subtypes in RECA-EU dataset. Figure S4: function enrichment analysis. A: BP of GO analysis. B: CC of GO analysis. C: MF of GO analysis. D: KEGG analysis. Figure S5: functional enrichment analysis. A: the GSEA revealed that tumor-associated pathways were correlated with RiskScore. B: 14 pathways and 13 pathways were inhibited in the high group than in the low group in the TCGA-KIRC cohort and RECA-EU dataset, respectively. [file 8446765.f1.zip › Figure S1 (1).jpg]

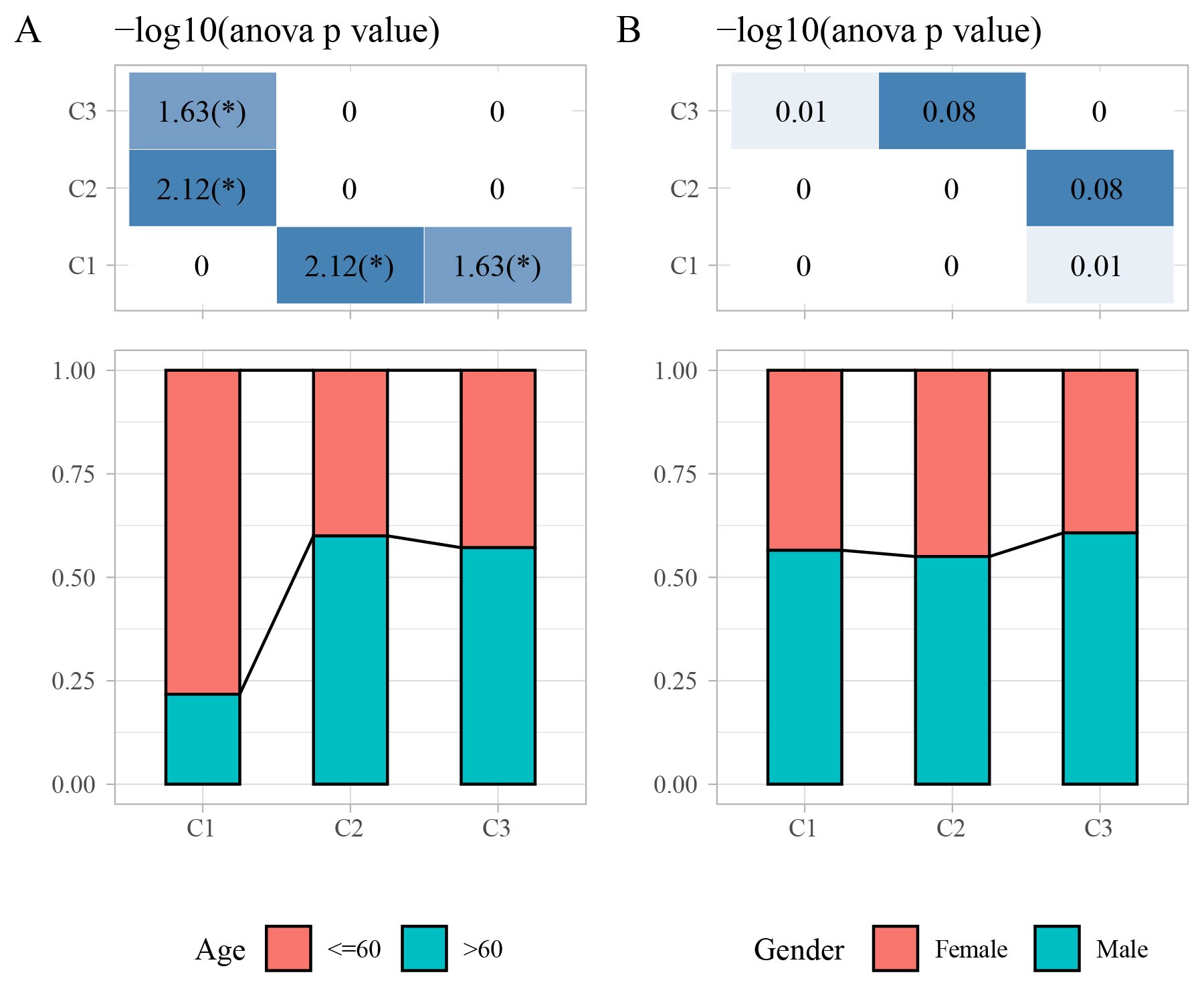

Supplement: Supplementary Materials — Figure S1: intersection analysis between TCGA-KIRC dataset and RECA-EU dataset to acquire necroptosis score positively related genes. Figure S2: the distribution of age (A), gender (B), grade (C), T stage (D), N stage (E), M stage (F), and stage (G) in three subtypes in the TCGA-KIRC dataset. Figure S3: the distribution of age (A) and gender (B) in three subtypes in RECA-EU dataset. Figure S4: function enrichment analysis. A: BP of GO analysis. B: CC of GO analysis. C: MF of GO analysis. D: KEGG analysis. Figure S5: functional enrichment analysis. A: the GSEA revealed that tumor-associated pathways were correlated with RiskScore. B: 14 pathways and 13 pathways were inhibited in the high group than in the low group in the TCGA-KIRC cohort and RECA-EU dataset, respectively. [file 8446765.f1.zip › Figure S3 (1).jpg]

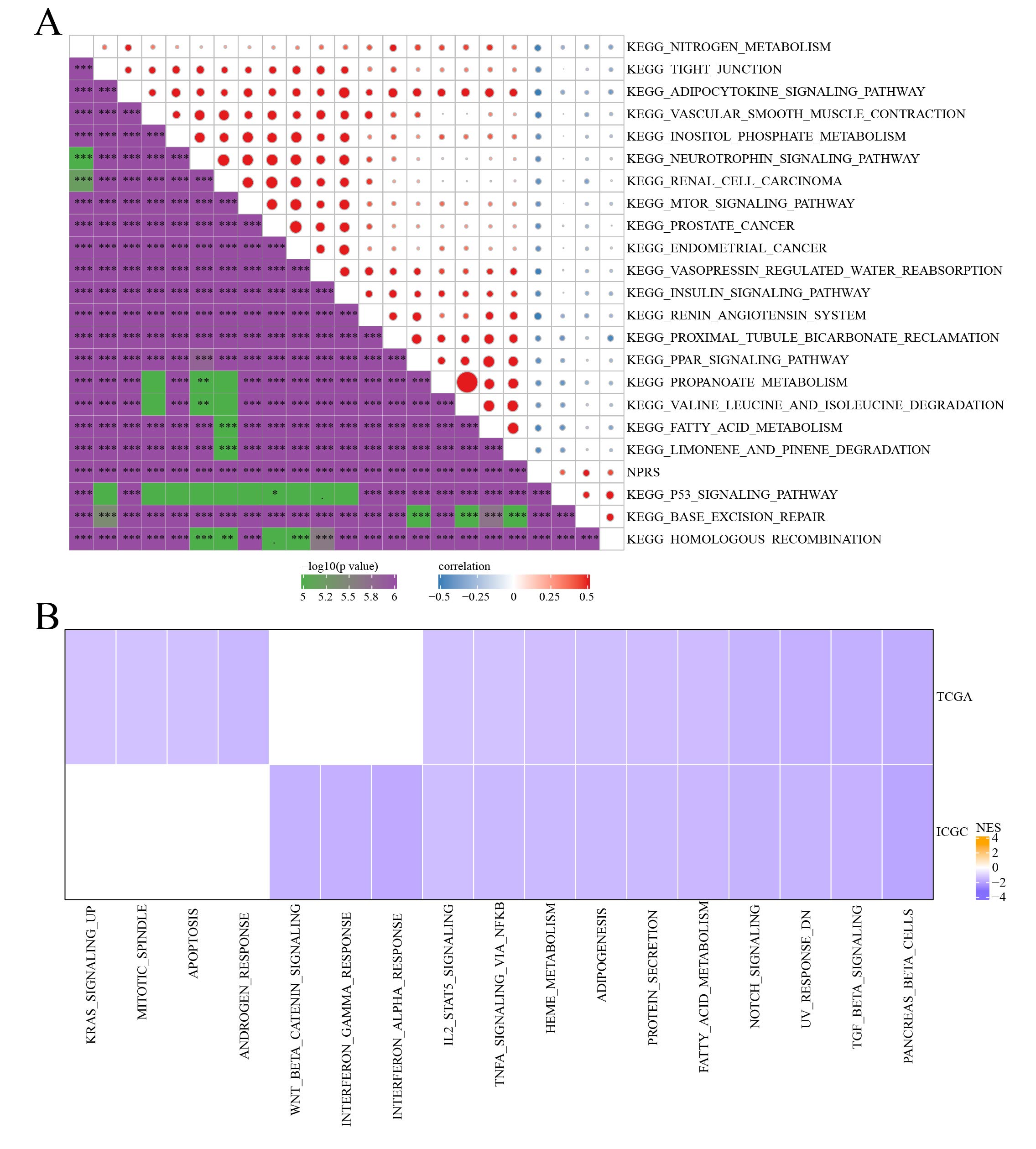

Supplement: Supplementary Materials — Figure S1: intersection analysis between TCGA-KIRC dataset and RECA-EU dataset to acquire necroptosis score positively related genes. Figure S2: the distribution of age (A), gender (B), grade (C), T stage (D), N stage (E), M stage (F), and stage (G) in three subtypes in the TCGA-KIRC dataset. Figure S3: the distribution of age (A) and gender (B) in three subtypes in RECA-EU dataset. Figure S4: function enrichment analysis. A: BP of GO analysis. B: CC of GO analysis. C: MF of GO analysis. D: KEGG analysis. Figure S5: functional enrichment analysis. A: the GSEA revealed that tumor-associated pathways were correlated with RiskScore. B: 14 pathways and 13 pathways were inhibited in the high group than in the low group in the TCGA-KIRC cohort and RECA-EU dataset, respectively. [file 8446765.f1.zip › Figure S5.jpg]
